# Expert Consensus on a Proposed Study Framework to Explore Factors Influencing *Plasmodium knowlesi* Malaria Preventive Behavior: A Modified Delphi Method Protocol

**DOI:** 10.3390/ijerph19074141

**Published:** 2022-03-31

**Authors:** Nurul Athirah Naserrudin, Rozita Hod, Mohammad Saffree Jeffree, Kamruddin Ahmed, Mohd Rohaizat Hassan

**Affiliations:** 1 Department of Community Health, Faculty of Medicine, Universiti Kebangsaan Malaysia, Kuala Lumpur 56000, Malaysia; drathirah85@gmail.com (N.A.N.); rozita.hod@ppukm.ukm.edu.my (R.H.); 2 Borneo Medical and Health Research Centre, Faculty of Medicine and Health Sciences, Universiti Malaysia Sabah, Kota Kinabalu 88400, Malaysia; saffree@ums.edu.my (M.S.J.); ahmed@ums.edu.my (K.A.); 3 Sabah State Health Department, Ministry of Health, Kota Kinabalu 88590, Malaysia; 4 Department of Public Health Medicine, Universiti Malaysia Sabah, Kota Kinabalu 88400, Malaysia; 5 Department of Pathobiology and Medical Diagnostics, Faculty of Medicine and Health Sciences, Universiti Malaysia Sabah, Kota Kinabalu 88400, Malaysia

**Keywords:** *Plasmodium knowlesi*, zoonotic malaria, Delphi study, modified Delphi study, decision making, expert consensus, consensus, preventive behavior

## Abstract

The increasing incidence of *P. knowlesi* malaria infection among humans is a public health threat. This zoonotic disease is challenging to eliminate owing to the presence of animal reservoirs. Understanding the factors such as the community’s belief, social context, drivers, and barriers can provide insights into malaria preventive behavior. It is crucial to improve the current preventive measures. This study aims to achieve consensus among malaria experts based on evidence from literature reviews and experts’ opinions on possible factors influencing malaria preventive behavior among communities exposed to *P. knowlesi* malaria infection. A modified Delphi study protocol was developed to gather experts’ consensus on the study framework to explore the factors influencing preventive behavior among communities exposed to *P. knowlesi* malaria infection. The framework is adapted from the ideation model, and it is integrated with other relevant theories and extensive literature reviews. We will use the modified Delphi protocol to reach a consensus. The experts will respond to each questionnaire item and a related open-ended questionnaire. Consensus is predetermined at more than 70% agreement on the items. We will use descriptive statistics and thematic analysis to analyze the data. All experts will remain anonymous to maintain the characteristics of a traditional Delphi study.

## 1. Introduction

The Delphi method originated in the early 1960s in a study developed by the Rand Corporation and sponsored by the United States Air Force, where it was first established as “Project Delphi”, anonymously [1]. The Delphi method gained popularity in the mid-1990s in military and non-military applications. The technique was described as valid for decision-making processes [2,3,4,5,6,7,8,9,10,11,12,13,14,15] and gradually applied in the healthcare sector [2,7,9,11,14,15]. Since then, it has been used as a technique to evaluate concepts and develop study frameworks [7,13,14], research justification, and theoretical processes [5].

The Delphi technique has been described as a qualitative data analysis technique [12,13]. However, each Delphi study requires rigorous measures—using both qualitative and quantitative inputs [10,11]. For the Delphi method to support a constructivist inquiry, the positivist criteria (objectivity, validity, and reliability) and trustworthiness (confirmability, credibility, transferability, and dependability) used in the qualitative research need to be applied [9,10]. However, various Delphi techniques have been used to define group consensus, expert selection, the number of rounds, and reporting on the method and results [4,5,8,9,10,11,12,13,14,15]. Therefore, researchers need to define “consensus” and the “cut-off value” if an agreement rate is used [8]. Although there are numerous challenges and questions raised when using this technique, it is essential for achieving consensus on issues where none existed previously [5].

A consensus is a general agreement or a unanimity of opinions of a predetermined group of experts [1,4,9]. Experts are knowledgeable, competent, and representative of the field of inquiry [13,14,15,16]. Such a consensus is considered most reliable because it depends on the controlled feedback of experts via a series of questionnaires [3,5,6,10]. The cut-off value, which defined as the value from which a consensus was made, can vary between 20 and 100% in health sciences literature [17]. A threshold of more than 60% was found in the majority of studies [17]. Some studies in the field of medicine define a consensus outcome when more than 70% of the participants are in agreement with the items presented in a questionnaire [13,14]. However, items that do not meet this criterion are either revised or discarded following a re-rating in a subsequent round of the study [13,14].

A Delphi method has some notable characteristics [2,5,6,7,8,9,10], such as the anonymity of the participants and controlled feedback [5,6,7,8,9,10]. However, since then, the traditional Delphi method has been modified by, for instance, using a structured questionnaire in the initial round [7,11] and including interviews or focus group discussions on supplementing the responses [10]. Participant anonymity is a primary characteristic and an advantage of the Delphi method, as it can reduce the effects of dominant individuals—a common concern when using group-based processes [2,9,11]. The issue of confidentiality is facilitated by the geographic dispersion of the participants along with the use of electronic communication [4,10,15].

Controlled feedback in the Delphi process reduces the effect of “noise”, which is described as communication that occurs in a group process that can distort the data, such as deals within the group or a focus on individual interests rather than group problem solving [4,8,15]. Additionally, controlled feedback allows participants to rethink prior answers and make changes based on the information and results provided [3,4,9,13,14,15,16]. It consists of a well-organized summary of prior iterations, which are intentionally distributed among the participants to generate additional insights and clarify the information obtained in the previous rounds. Through multiple iterations, participants are expected to become more oriented toward problem-solving, offer more insightful opinions, and minimize the effects of noise [4,15]. The use of various statistical techniques further reduces the potential of group pressure for conformity [4,8,10]. Specifically, statistical analyses can ensure that opinions generated by each participant in a Delphi study are well represented in the final iteration because there may still be a significant divergence in individual opinions at the end of the round [1].

This study aims to achieve consensus among malaria experts based on empirical evidence from literature reviews and the experts’ opinions on possible factors influencing malaria preventive behavior among communities exposed to *P. knowlesi* malaria infection.

## 2. Methods and Analysis

### 2.1. Study Aim

We address the following research question: What factors should be included in the study framework to guide the exploration of factors contributing to zoonotic malaria preventive behavior in communities exposed to *P. knowlesi* malaria? The results of the study are expected to help form a study framework to guide future *P. knowlesi* studies in exploring the factors influencing the malaria preventive behavior among exposed community to this zoonotic infection and strategize future zoonotic malaria control measures. We employ the Delphi method to achieve this goal and reach a consensus on items that, according to the participants, who are malaria experts, can facilitate exploration of the possible contributing factors.

### 2.2. Procedure

This study employed a pragmatic approach [2,8,15,17]. Previous studies have used the traditional and modified Delphi approach to reach a consensus on their study framework and health management procedures [7,14,16,18,19]. The research methods were established by the co-authors, who are experts in the fields of epidemiology, infectious disease, and social science. The author and co-authors had virtual meetings and email communication, in which an agreement was reached regarding the inclusion and exclusion criteria for the study’s participants. The study framework was discussed to integrate different models and design the draft questionnaire. Once the questionnaire is validated, participants will be recruited for the pilot study, and another group will be recruited for the definite Delphi study. Descriptive analysis will be conducted using Statistical Package for Social Science (SPSS) for the Windows version 26 (SPSS Inc., Chicago, IL, USA) [20]. The thematic analysis method by Braun and Clarke will be used for the open-ended response [21].

Internet technologies, such as email and Google forms, will facilitate the Delphi procedure and aid the study [4,7,9,11,12,13,15,22]. Moreover, Google Drive (Google LLC, Mountain View, CA, USA) will be used to store the research proposal, study-related documents, malaria literature, and all the related information in further rounds between the researchers and participants. Inputs from panelists were stored on online data storage drives [7]. Furthermore, it is more practical to use electronic and online modes for the Delphi procedure to eliminate the need for travel, allow for the recruitment of geographically dispersed experts, communicate with the experts more conveniently, and increase efficiency in data collection [4,7,9,11,12,13,15,22]. Based on a review paper by Niederberger and Spranger [17], as there is no standard technique for Delphi study, the Standard for Conducting and Reporting Delphi Studies (CREDES) will not be considered.

The number of rounds is determined a priori to avoid false consensus and participant fatigue, which can result in a premature agreement to end the process [9,10]. The estimated completion period for the Delphi study is five months, with each round lasting four weeks. After the initial invitation, a reminder will be sent, approximately every seven days [13], to track the responses [10,13,15]. Scholars generally recommended two to three rounds [2,3,8,10,13,14,17]. Accordingly, we opted for three rounds, as this is considered optimal [14,15,23]. The criteria for terminating the Delphi process and achieving consensus were predetermined as greater than 70% agreement on the questionnaire items [17].

We will employ a structured questionnaire based on the possible factors impacting malaria preventive behavior. These will be used as items in the proposed study framework. The questionnaire will include items for the theories, comprising of knowledge, beliefs, environmental barriers and drivers, and attitudes that contribute to *P. knowlesi* malaria preventive behavior. These factors were identified based on the literature and systematic reviews undertaken by the co-authors and presented at a recent conference. We assume that human behavior is influenced by both internal factors, such as the background and attitude, and external factors, such as environmental drivers and barriers. Thus, the proposed conceptual framework is adapted according to the ideation model of communication and behavior change [24], the explanatory model of Arthur Kleinman [25], and Murdock’s model of theories of illness causation [26]. The ideation model has been used to guide the evaluation of health communication intervention and strategic design of a health program [24].

The initial challenge in developing the questionnaire is defining the questions to obtain accurate and valuable feedback from experts. The questions have to be concise and direct, have a logical connection, and encompass the required information without being too time-consuming for the respondents [4,5,8]. We have created three headings (or domains) based on the three models, under which all related items in each model were included. On the basis of this initial items pool, we formulated the Round 1 questionnaire. We will use structured questions, for example: “Should this item be included to explore the factors influencing malaria preventive behavior in communities exposed to *P. knowlesi* infection?”. The participants will be given the opportunity in Round 1 to add items based on their opinions to generate more comprehensive items for the study framework. Each survey is estimated to take 20–40 min to complete. Participation in iterative rounds allows participants to review their own and collective answers before finally submitting their responses [4,10,14,16,17]. The participants will answer the questionnaire on a 5-point Likert scale, ranging from 1 (strongly disagree) to 5 (strongly agree) [3,16,18]. The ratings will be grouped into three levels: low score (1–2), average score (3), and high score (4–5) [18,27]. In addition to quantitative responses, participants will also be allowed to provide comments or justify their choices on the items (qualitative data) [3,4,5,9,12,15,16,27]. They will be asked to provide recommendations or suggestions regarding any additions or deletions to the list of proposed items in the questionnaire. A consensus is defined as greater than 70% [17] of the participants agreeing (answer “3”), strongly agreeing (answers “4” or “5”), or disagreeing or strongly disagreeing (answers “1” or “2”) with a given item [13,14]. This will allow the ordinal data to be described using percentages of responses in each category. In addition, median will also be measured to achieve the consensus on the items. The employment of a median to measure the central tendency from ordinal data has been used by some researchers to reflects the convergence of opinion because the distribution of ratings is generally skewed [4]. A higher median suggests a greater degree of consensus [4].

### 2.3. First Step: The Problem Statement

**Figure 1:**
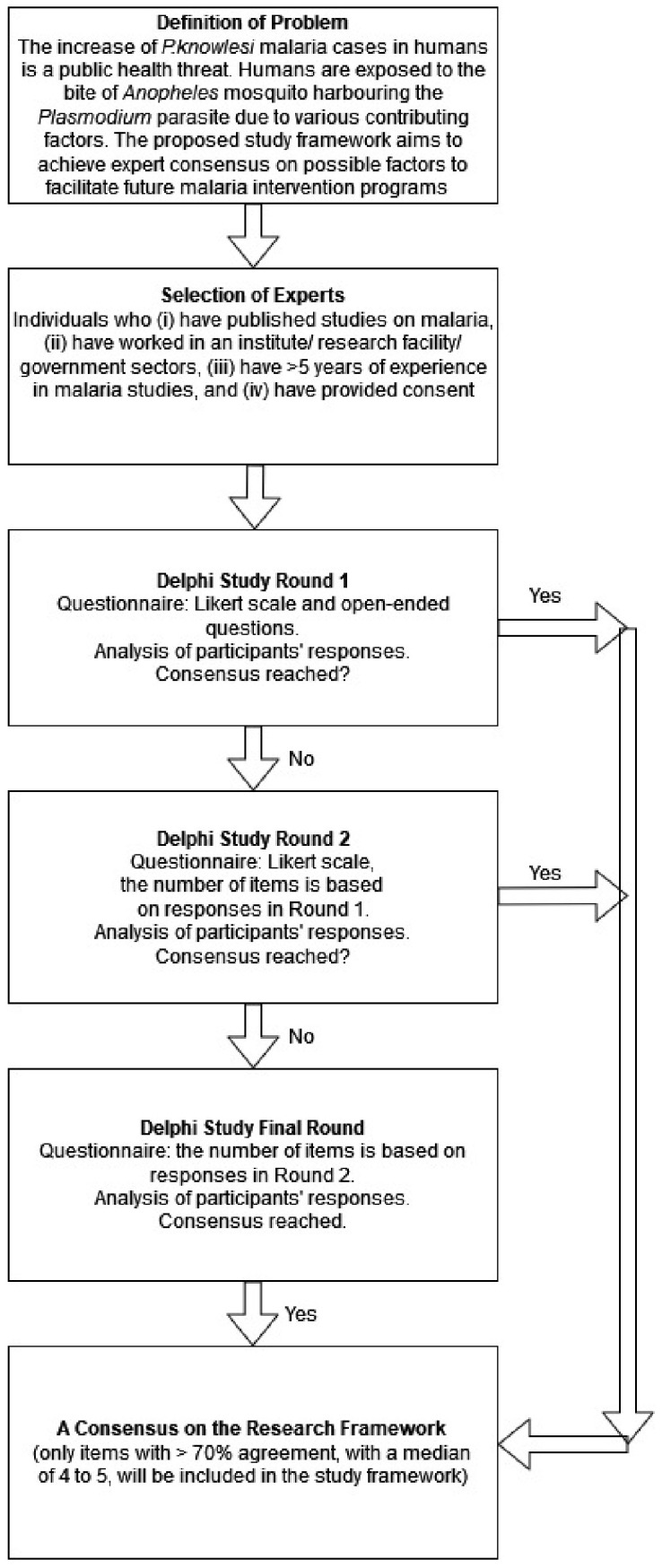
The steps in the modified Delphi study for evaluating the proposed study framework.

The first step is to explain the research problem and define the questionnaire to be sent to the experts [3,9,10,21] (Figure 1). The threat of *P. knowlesi* malaria to humans has received significant attention in the World Malaria Report [28], in discussions among malaria experts in the Evidence Review Group on *P. knowlesi* malaria [29], and from the World Health Organization’s (WHO) Malaria Policy Advisory Group (MPAG) [30]. In the last few decades, the increasing number of *P. knowlesi* malaria cases in human has become a public health threat [29,30,31,32]. Its widespread geographical distribution in South East Asia [33], the disease burden presented by various disease outcomes from asymptomatic infections (that can only be detected by using more sensitive and specific molecular screenings), and severe disease and reported fatalities strongly indicate the need for future studies to improve our understanding of *P. knowlesi* malaria [29,30,34,35]. This is further compounded by the fact that it is a complex disease that is challenging to control [28,29,30]. Exposure to infection is highly significant among agricultural workers, males, adults, and individuals working near forests [29,30,31,32]. The modeling of malaria is arduous as the *Plasmodium* parasite co-evolves with the vertebrate host to survive [36]. The present study focuses on human behavior as the affected host. Understanding human behavior and social aspects in malaria control could help sustain the health program [37]. Human behavior is complex, as it encompasses daily life and is influenced by various factors [37]. Given the need to explore the social context, attitude, and other contributing factors that can influence human behavior, it is essential to build a study framework to explore these possible factors of malaria preventive behavior. The framework could be a theoretical lens to facilitate social and behavior change programs toward a sustainable malaria control program.

Based on an extensive review of the literature on malaria, a study framework was designed as a theoretical guide for future studies. Two systematic reviews were conducted to identify the vulnerability factors of *P. knowlesi* malaria infection among human subjects (registered under PROSPERO: CRD42021243374). The first systematic review identified the background factors influencing the risk for *P. knowlesi* malaria, including being male, adult, plantation workers, living near the forest, and history of traveling to the forest. In contrast, the second systematic review identified the human behavior, perspectives, and activities at the risk of exposure to *P. knowlesi* malaria. This systematic review was reported as an oral presentation in the recent International Conference of Public Health 2021 (https://drive.google.com/file/d/1Sn5lUw-NJbh-BA-S5JO-E9mSkIMQJgnU/view), accessed on 15 August 2021. It has also been registered under Prospero: CRD42021251323.

**Figure 2:**
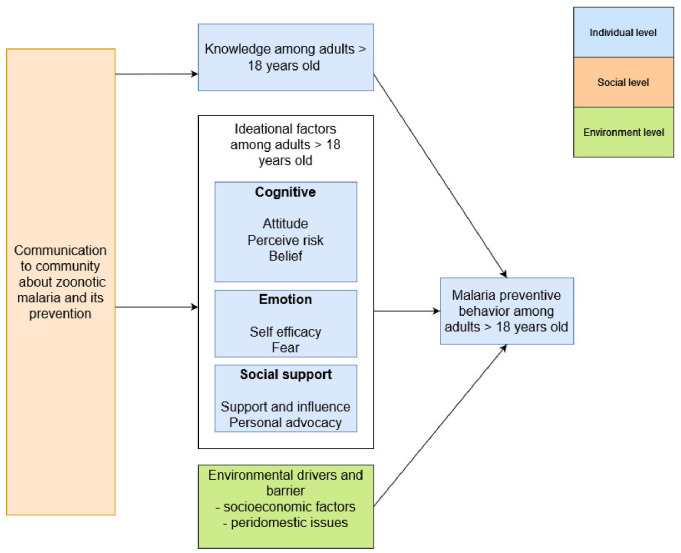
The proposed study framework.

We propose a metatheory to answer the research question. We propose the use of the ideation model to identify the factors that predict behavior [24,38,39]. It is an evidence-based predictive model for behavior change [24,38,39]. Behavior is influenced by multiple social and psychological factors and environmental conditions that facilitate behavior [24,38,39]. Ideation factors are grouped into three categories: cognitive, emotional, and social. These elements combine to affect behavior, similar to how multiple risk factors affect the probability of contracting a disease [24]. When the ideation is used in programs, researchers measure the factors that apply to a person and then combine them to create an ideation score that predicts how likely a person is to adopt a behavior [24]. The higher it applies to someone, the greater their likelihood of adopting a healthy behavior [24]. When ideational factors are aggregated in this way, they are highly predictive of health behaviors [24,38,39]. We integrate Murdock’s model of local illness belief in our framework, as it allows for a deeper exploration of community beliefs related to illness, caused either by supernatural or natural causes [26]. The emic of disease etiology, symptoms, and treatment differs from medical professionals and is conceptualized by the explanatory model [25]. The proposed study framework is shown in Figure 2.

### 2.4. Second Step: The Selection of Experts

In our study, the recruitment of experts commenced in 8 August 2021 after the ethics board approved the study. In our Delphi study, we identified experts after an extensive literature review and peer-supervisory recommendations [21,27]. We used purposive and snowball sampling [13] to recruit academia and the public sector [7,8,10]. There is no consensus in the literature on the optimal number of participants in a Delphi study. Twelve or more participants is effective, while less than six participants are generally considered insufficient [9]. Thus, we opt for a sample size of 12, mainly as the Delphi study depends on group dynamics to achieve consensus [13].

The participants must meet the following inclusion criteria: (1) individuals who have published one or more studies on malaria in peer-reviewed journals, (2) individuals who have more than five years’ experience in malaria studies or management, and (3) individuals who are currently working or practicing in any research institution or have an administrative post in an organization. The sample will include individuals from different professions, such as researchers, lecturers, professors, directors, and the heads of departments of different organizations worldwide. Candidates who do not indicate an interest in participating in the modified Delphi study after receiving two emails within a two-week period will be excluded from the study. Potential participants will be contacted via email, informing them about the study, and will be given an overview of the modified Delphi study, its objectives, phases, and their commitment to reach consensus. Each expert will be sensitized to the objectives and methodology of the study by sharing the research proposal and providing assurance of anonymity. Furthermore, upon receiving the experts’ consent to participate in the study, a formal e-consent will be obtained to complete the recruitment process. All data will be stored in a password-encrypted computer in a locked office, following standard guidelines. The data will be destroyed after five years, as per standard guidelines [13].

### 2.5. Third Step: Round 1 of the Study

#### 2.5.1. Questionnaire Validation

Before Round 1 commences, we conducted a questionnaire validation that included a content validation process and was face-validated by six English-speaking participants to ensure the questionnaire’s quality [40] (see Supplementary Questionnaire S1 for questionnaire draft). The questionnaire validation allows further modification, clarification, and augmentation to fit the study’s objectives [40] (see Text S1 for participants’ responses and opinions on the draft). After completing the questionnaire validation phase, a pilot study will be conducted. A pilot study is relatively uncommon in published Delphi studies [41]; however, we aim to ensure that our study has the necessary methodological rigor to produce high-quality research.

#### 2.5.2. Pilot Study

A pilot study helps ensure questionnaire clarity before it is distributed to the Delphi participants by allowing comments from respondents and improvements based on their comments [41]. Thus, feedback from the pilot study is essential for suggestions regarding the retention or omission of items [41]. As suggested by Clibbens et al. [41], we propose adopting an approach of purposive and convenience sampling for the pilot study. A sample is purposive when the volunteers meet some of the predefined inclusion criteria of the complete study. Convenience sampling is employed when each volunteer is already in contact with the researcher(s) through a university. Seven people with experience in conducting malaria studies will be recruited for this pilot study. By using such a method for the pilot study, the participants may have a stronger motivation to respond. The output of the pilot study will be evaluated by the research team before conducting Round 1 of the modified Delphi study [42] (see Supplementary Questionnaire S2 for the pilot study questionnaire).

#### 2.5.3. Round 1

In a traditional Delphi study, Round 1 begins with an open-ended questionnaire. In the modified Delphi study, we propose the use of a literature review, a structured questionnaire, and open-ended questions to justify responses and share opinions from the malaria experts (participants) [3,4,5,9,12,15,16]. In Round 1, data on participant demographics will be collected, including gender, location of current work, current job position, and the number of years in the field of malaria research. The experts will also be allowed to provide input on other items that should be included in the study framework. The responses collected in Round 1 will be analyzed individually and collectively. By using descriptive statistics and thematic analysis, the responses will be shared iteratively in the following rounds [2,5,9,10]. Only items that reach a consensus of >70% agreement with a median of 4 to 5 will be kept for Round 2.

### 2.6. Fourth Step: Round 2 of the Study

Only participants who complete their responses in Round 1 will be invited to participate in Round 2. In Round 2, each Delphi participant will receive a questionnaire by email and be asked to review the responses provided in Round 1, as summarized by the researcher; participants will also be able to revise their initial responses [5,13,14]. The results will include the overall score for each item using a measure of central tendency, median, and the percentages of each item. The open-ended questionnaire will be analyzed using thematic analysis and shared as a supplementary file to ensure data trustworthiness and the credibility of the study [12]. Hence, the results of Round 2 could be lengthy and detailed [8,12,13,14]. All items that do not reach a consensual median score of at least 3 will be discarded in Round 2 [13,14]. There will be no option for a free-text response (open-ended questions) in Round 2. A consensus will be reached, and the items will be evaluated based on the group’s collective responses. The responses that reach an agreement of >70% [17] with a median of 4 to 5 will be retained [21].

### 2.7. Final Step: Round 3

In Round 3, the consensus stage, each participant will receive an individualized and collective response comprising only items with an agreement of greater than 70% [17] and a median of 4 to 5 [21]. Three Delphi rounds—considered optimal to reach a consensus—were predetermined to enable an adequate reflection on the group’s responses [14,15,23]. The consensus is the “general agreement of a substantial majority” of the Delphi participants [27] of the items proposed by the researcher and the experts to be included in the study framework.

### 2.8. Current Status

The questionnaire validation process commenced on 20 August 2021. The draft questionnaire was distributed to a different subgroup of malaria experts. Upon completion of the questionnaire validation, the questionnaire were distributed to pilot study participants. Subsequently, the Round 1 Delphi will be conducted upon the completion of pilot study analysis with a different subgroup of malaria experts, different from the participants for the questionnaire validation. A paper reporting this protocol will be submitted for a conference presentation, and once the original results are available, the study will be submitted for publication in 2022.

### 2.9. Patient and Public Involvement

There is no patient or public involvement in this modified Delphi study.

## 3. Discussion

The modified Delphi study will produce a framework that will act as a theoretical lens for future studies related to malaria preventive behavior in communities exposed to *P. knowlesi* malaria infection. There are currently known various contributing factors in a community that could impact the malaria preventive behaviors such as the emotion, attitude, and environmental challenges [24].

The adaptive framework of the ideation model is evidence-based and has been used in family planning [39], HIV/AIDS intervention, and during Ebola outbreaks [38]. However, it is yet to be adapted in zoonotic malaria programs. There is a positive relationship between high levels of ideation and behavior change; thus, it is a valid model to integrate into our study. The model can identify individual community factors relating to preventive measures that should be addressed in a strategic communication program [24,38,39]. It can also be used to evaluate interventions and verify the effectiveness of a strategy. In this way, the model can help tighten the links between strategic planning and program design in zoonotic malaria control. The implementation can result in more effective and sustainable communication activities in the targeted community and ultimately improve health outcomes. A comprehensive framework for the prevention and control of vector-borne diseases such as malaria can provide guidance on planning and coordinate strategic, efficient, and multisectoral collaboration and the roles and responsibilities of different stakeholders. It can help governments understand how nongovernmental stakeholders and communities can be engaged in the process of disease control [42]. The study’s findings will be disseminated in peer-review publications, conference presentations, and propagated among policymakers.

## 4. Conclusions

This modified Delphi method protocol presents an overall picture of how the study will be conducted. The application described its detailed strategies to address the research questions on possible factors influencing malaria preventive behavior among communities exposed to *P. knowlesi* malaria infection—from the opinions of malaria experts. Ensuring the consensus among these experts can provide novel contributions for a more effective strategy to control the disease in providing guidance to future exploratory study in the communities.

## Data Availability

Data available on request from the corresponding author.

## Supplementary Materials

The following supporting information can be downloaded at: https://www.mdpi.com/article/10.3390/ijerph19074141/s1, Supplementary Questionnaire S1: The questionnaire draft, Supplementary Questionnaire S2: The pilot study questionnaire, Supplementary Text S1: Participants’ response and opinion on the draft.
