# Supplementary material for: Expert Consensus on a Proposed Study Framework to Explore Factors Influencing Plasmodium knowlesi Malaria Preventive Behavior: A Modified Delphi Method Protocol
Source: Int J Environ Res Public Health. 2022 Mar 31;19(7):4141. doi: 10.3390/ijerph19074141 (PMC8998657; doi:10.3390/ijerph19074141)
Supplement: Supplementary file 1 [file ijerph-19-04141-s001.zip › ijerph-1575192-supplementary.pdf]

Supplementary materials

Supplementary Questionnaire S1: The questionnaire draft

Supplementary Questionnaire S2: The pilot study questionnaire

Supplementary Text S1: Participants’ response and opinion on the draft

Supplementary Questionnaire S1: The questionnaire draft

No	Question	Extremely disagree (1)	Disagree (2)	Neither disagree or agree (3)	Agree (4)	Extremely agree (5)	I do not know
**1.Demographic background**							
1.1	**Age** could influence individual behaviour and exposed him/her to *P. knowlesi* infection						
1.2	**Gender** could influence individual behaviour and exposed him/her to *P. knowlesi* infection						
1.3	**Ethnicity** could influence individual behaviour and exposed him/her to *P. knowlesi* infection						
1.3	**The marital status** could influence individual behaviour and exposed him/her to *P. knowlesi* infection						
1.4	Based on the **demographic background,** would you like to explain why you choose the scale for the respective questions?						
**2. Socioeconomic status**							
2.1	**Education level** could influence individual behaviour						

	and exposed him/her to *P.knowlesi* infection						
2.2	**The occupation** could influence individual behaviour and exposed him/her to *P.knowlesi* infection						
2.3	**The house income** could influence individual behaviour and exposed him/her to *P.knowlesi* infection						
2.4	The **housing condition** could expose an individual to *P.knowlesi* infection						
2.5	The **peridomestic housing condition** could expose an individual to *P.knowlesi* infection						
2.6	Based on the questions related to socioeconomic status, would you like to explain why you choose the scale to the respective questions?						
3	The Ideation Model						
	Note<br>1.The model is used by the Strategic Communication and Behaviour Change Programme to help in design a specific intervention program. It emphasizes the importance of effective communication for behaviour change. It has specific ideation factors which are grouped into three categories: **cognitive, emotional, and social support**. These are all psychosocial						

	factors that can predict behaviour. Based on the Ideation Model, behaviour is also influenced by **knowledge and skills and environmental conditions** that facilitate the behaviour.<br>• **Cognitive factors**: the individual's beliefs, values, and attitudes, subjective norm (perceive about others thinking), and social norms (thinking about what others are doing)<br>• **Emotion** describes how the individual feels about the behaviour and how confident the person is to perform it.<br>• **Social factors**: this describes the interpersonal interaction or support from the surrounding people<br>2. An individual is more likely to adopt or perform the behaviour if more ideational factors are applied. The summation of ideational factors is highly predictive of health behaviour.						
3.1	**Cognitive factors** could influence individual behaviour and exposed him/her to *P.knowlesi* infection						
3.2	**Emotion** could influence individual behaviour and exposed him/her to *P.knowlesi* infection						
3.3	**Social factors** could influence individual behaviour and exposed him/her to *P.knowlesi* infection						
3.4	**Knowledge** could influence individual behaviour and exposed him/her to *P.knowlesi* infection						
3.5	**Environmental factors (including socioeconomic factors)** could influence individual behaviour and						

	exposed him/her to *P.knowlesi* infection						
3.6	**Human behaviour** could expose a person to *P.knowlesi* infection						
3.7	Based on the questions related to the Ideation Model, would you like to explain why you choose the scale for the respective questions?						
4	Murdock's Model; The aetiology of illness (1978)						
Note.<br>The model was based on the worldwide survey on illness. It conceptualizes the beliefs about ill-health causation within the community. It is either caused by **natural causation (infection or accident) or supernatural causation (including mystical causes, animistic causes, and magical causes).**							
4.1	The model of illness causal being **supernatural or natural causes** could influence individual behaviour and exposed him/her to *P.knowlesi* infection						
4.2	Based on the questions related to Murdock's Model of illness aetiology, would you like to explain why do you choose the scale to the respective question?						
6	The Explanatory Model (EM) by Arthur Kleinman (1978)						

	1.The model explained that patients' and the close person who experience the illness are influenced by social and cultural factors and could be described narratively. This differs from the doctor who treats the "disease" from medical and scientific logic.<br>2. The model focuses on how communication can help explain a patient's experience, which is far more complex and deeply interconnected with their daily lives.						
6.1	The Explanatory Model (Kleinman 1978) could support the study to explore the sociocultural belief and psychosocial factors that could influence individual behaviour and exposed him/her to *P.knowlesi* infection.						
6.2	Based on the questions related to the Explanatory Model, would you like to explain why do you choose the scale to the respective question?						
7	Expert's opinion						
7b	In your opinion, what are the other variables that can be included or excluded to improve the research framework ?<br>**You can give more than 1 answer.**						

Supplementary Questionnaire S2: The pilot study questionnaire

Demographic profile:

1. What is your gender?
2. In which institution are you working now?
3. How long have you been doing studies or working in the malaria field (in years)?
4. What is your position in your institution or working area?

	Question	Extremely disagree (1)	Disagree (2)	Neither disagree or agree (3)	Agree (4)	Extremely agree (5)	I do not know
**1. Demographic background**							
The demographic background is the factor that could influence malaria preventive behavior and expose the individual/ community to Plasmodium knowlesi malaria infection. For example, the preventive behaviour is measures such as wearing protective clothing while performing outdoor work, putting on Insecticide Treated Nets (ITNs) or Long Lasting Insecticide Treated Nets (LLINs) during sleep inside the house or in the farm and not performing outdoor work after dark							
1.1	**Age** could influence the malaria preventive behavior and exposed an individual to *P. knowlesi* malaria infection						
1.2	**Gender** could influence the malaria preventive behavior and exposed an individual to *P. knowlesi* malaria						
1.3	**Occupation** could influence the malaria preventive behavior and exposed an						

	individual to *P. knowlesi* malaria						
1.4	**Ethnicity** could influence the malaria preventive behavior and exposed an individual to P. knowlesi malaria infection						
1.5	Based on the demographic background, would you like to explain why do you choose the scale, to the respective questions??						
2	The Ideation Model						
	The Ideation Model (Center for Communication Programs, John Hopkins University). The model is a metatheory model that emphasizes the importance of effective communication for behavior change.<br><br>The study will explore the ideation factors (cognitive, emotional, and social support) among individuals and communities exposed to P. knowlesi malaria, which could facilitate the design of a specific intervention programme based on the local context. They are more likely to adopt or perform the malaria preventive measures if more ideational factors are present.						
2.1	**Cognitive factors** could influence the malaria preventive behavior and expose an individual to *P. knowlesi* malaria infection<br><br>Cognitive factors describe the individual's beliefs, values and attitudes, subjective norms						

	(perceive others' thinking) and social norms (thinking about what others are doing). For example: How does a person/ community believe a person can get the infection through mosquito bites and not sleeping under the bed net?						
2.2	**Emotion** could influence the malaria preventive behavior and expose an individual to P. knowlesi malaria infection<br><br>Emotion describes how the individual feels about the behaviour and how confident the person is to perform it. For example: How does a person feel for wearing protective clothing while going hunting in the forest?						
2.3	**Social supports** could influence the malaria preventive behavior and expose an individual to *P. knowlesi* malaria infection						

	Social supports describe the interpersonal interaction or support from the surrounding people. For example: How does community support towards neighbours by staying indoors after dark facilitates the behavior to avoid being bitten by a mosquito						
	Besides the ideational factors, in the Ideation Model, the individual and community's knowledge, skills, and environmental conditions could also facilitate or causing barrier to the behavior change						
2.4	**Knowledge** could influence the malaria preventive behavior and expose an individual to P. *knowles*i malaria infection<br><br>Knowledge describes what the individual and community understand about the disease, transmission, activities at risk, and protective measures such as wearing protective clothing when performing outdoor work and						

	putting on bednets during sleep.						
2.5	**Environmental factors** could influence the malaria preventive behavior and expose an individual to P. knowlesi malaria infection<br><br>The environmental factors are extrinsic factors that may influence the malaria preventive measures such as education level, occupation, duration to work, frequency of going to work in the jungle, travelling to work, household income, home condition, and peridomestic condition. For example, the peridomestic condition is the surrounding area of the individual house, like long grass, paddy fields, water ponds, oil palms, macaque monkeys and others. The home condition is described as the house characteristic. For example,						

	"village-houses" are commonly built by rattan or bamboo, present open eaves on the wall, and provide ways for the mosquito to fly indoors.						
2.6	Based on the questions related to the Ideation Model, would you like to explain why you chose the scale to the respective questions?						
3	Murdock's Model; The theory of illness (1978)						
Murdock's Model; The theory of illness (1978). The model was based on the worldwide survey on illness causal. It conceptualizes the beliefs about ill-health causation within the community, either caused by natural (such as infection or accident) or supernatural causation (included mystical causes, animistic causes, and magical causes). It also acknowledges how a community perceives the causes of illness.							
3.1	The individual or community beliefs on the natural or supernatural causation of malaria could influence the malaria preventive behavior and expose an individual to *P. knowlesi* malaria malaria						

3.2	Based on the questions related to the Murdock's Model of illness aetiology, would you like to explain why you chose the scale to the respective question?						
4	The Explanatory Model (EM) by Arthur Kleinman (1978)						
	The Explanatory Model by Arthur Kleinman (1978). The model conceptualizes how communication can help to understand a patient's, family members or relatives' experience about the disease etiology, beliefs, meaning, symptoms, treatment-seeking behavior, and adaptation to the disease through their own cultural variations. Questions such as "What causes the disease" or "How do you think you were infected" will be asked to research participants. It is far more complex and deeply interconnected with their daily lives.						
4.1	The Explanatory Model (Kleinman 1978) will facilitate the exploration of the individual and community factors that influence the malaria preventive behavior of the community exposed to *P. knowlesi* malaria.						
4.2	Based on the questions related to the Explanatory Model, would you like to explain why you chose the scale to the respective question?						
5	Expert's opinion to improve the study framework						

7b	In your opinion, what are the other factors that should be included or excluded in exploring the influence of zoonotic malaria preventive behavior in individuals or communities exposed to P. *knowlesi* malaria infection?<br><br>You can provide more than one answer		

Supplementary Text S1: Participants' response and opinion on the draft

1. **Demographic factors**
   - We have excluded the **marital status** in the review as suggested by experts.
   - We have modified the **"behavior" to "malaria preventive behavior"** to give a more explicit statement on the study focus, which is to explore the malaria preventive behavior in the community. We have provided a brief note before prior to the demographic question: "In this study, the demographic background (age, gender and ethnicity) will be included as the background factors that could influence the malaria preventive behavior and exposed the individual/ community to *Plasmodium knowlesi* malaria (eg: wearing protective clothing while performing outdoor work, putting on bed nets during sleep inside the house or in the farm, not performing outdoor work after dark)".
   - The age, gender, and ethnicity word were kept as it is, as we will explore more during the exploratory phase of the study (Phase 2).
2. **Socioeconomic status**
   - The socioeconomic status was found to be redundant as the Ideation Model also includes the socioeconomic status in the environmental factors that may influence the behavior.
   - Descriptions were also provided for peridomestic condition, and home condition.
3. **The Ideation Model**
   - We have provided more description in the Ideation Model as experts commented the words used in the questionnaire draft were too general and causing difficulty to understand the meaning.
   - Examples for each Ideation factor are included based on the comments
   - Experts have commented to include knowledge in the questions. The Ideation Model does include knowledge as one of the factors that influence behaviour.
   - Experts have commented to include the duration to go to work and the frequency of working in the jungle. This question will be asked during the next phase of the study (exploratory study)
   - A diagram is included to provide a better view of the Ideation Model (Figure 1.0)

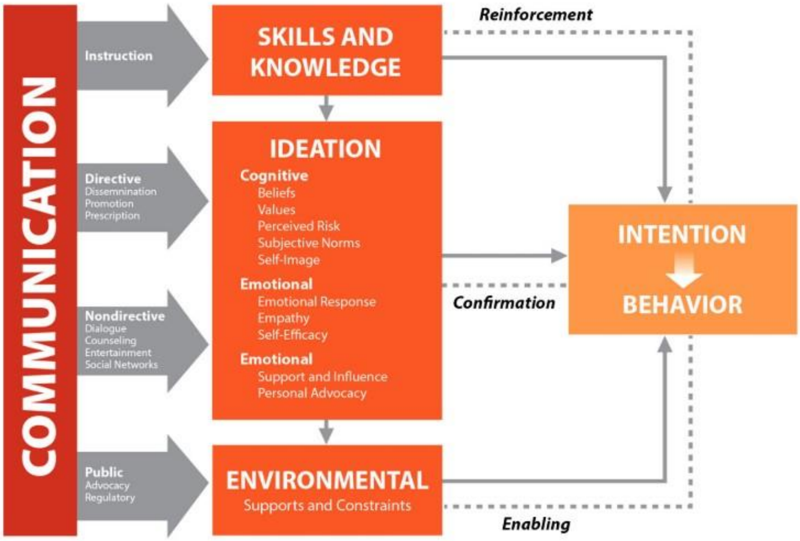

Figure 1.0 The Ideation Model

4. The theory of illness by Murdock (1978)

- A diagram is included to provide a better view of the theory (Figure 2.0)
- In some communities, the belief of the illness aetiology influences their malaria preventive behavior and perceived threat of the disease. We will explore this in the exploratory phase of the study.

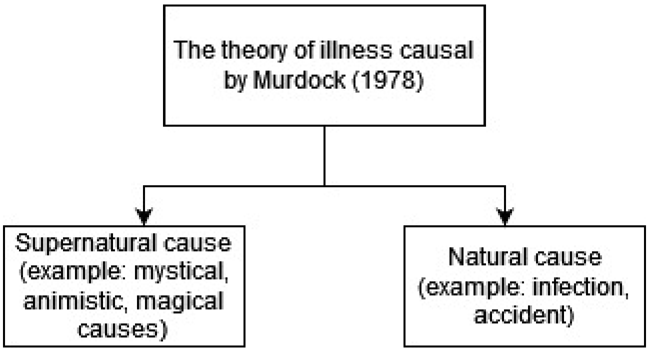

Figure 2.0 The theory of illness causal by Murdock (1978(

5. The Explanatory Model

- A diagram is included to provide a better view of the theory (Figure 3.0)

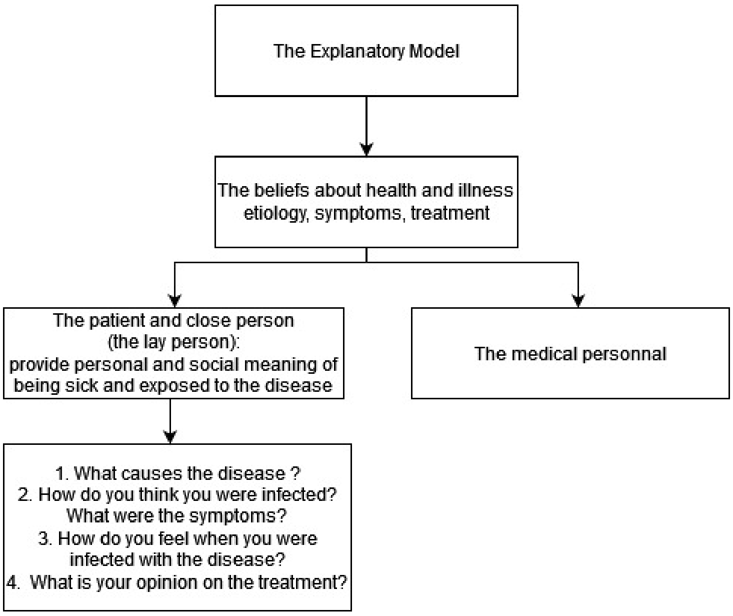

Figure 3.0 The Explanatory Model
